# Disseminating child abuse clinical decision support among commercial electronic health records: Effects on clinical practice

**DOI:** 10.1093/jamiaopen/ooad022

**Published:** 2023-04-13

**Authors:** David A Feldstein, Isabel Barata, Thomas McGinn, Emily Heineman, Joshua Ross, Dana Kaplan, Francesca Bullaro, Sundas Khan, Nicholas Kuehnel, Rachel P Berger

**Affiliations:** Department of Medicine, University of Wisconsin School of Medicine and Public Health, Madison, Wisconsin, USA; Department of Emergency Medicine, Donald and Barbara Zucker School of Medicine at Hofstra/Northwell, Hempstead, New York, USA; CommonSpirit Health, Chicago, Illinois, USA; Department of Medicine, Baylor College of Medicine, Houston, Texas, USA; Department of Pediatrics, UPMC Children’s Hospital of Pittsburgh, Pittsburgh, Pennsylvania, USA; Department of Emergency Medicine, University of Wisconsin School of Medicine and Public Health, Madison, Wisconsin, USA; Department of Pediatrics, University of Wisconsin School of Medicine and Public Health, Madison, Wisconsin, USA; Department of Emergency Medicine, Donald and Barbara Zucker School of Medicine at Hofstra/Northwell, Hempstead, New York, USA; Department of Emergency Medicine, Donald and Barbara Zucker School of Medicine at Hofstra/Northwell, Hempstead, New York, USA; Department of Medicine, Baylor College of Medicine, Houston, Texas, USA; Center for Innovations in Quality, Effectiveness and Safety, Michael E. DeBakey Veteran Affairs (VA) Medical Center, Houston, Texas, USA; Department of Emergency Medicine, University of Wisconsin School of Medicine and Public Health, Madison, Wisconsin, USA; Department of Pediatrics, UPMC Children’s Hospital of Pittsburgh, Pittsburgh, Pennsylvania, USA

**Keywords:** child maltreatment, physical abuse, electronic health record, clinical decision support, Epic, Allscripts

## Abstract

**Objectives:**

The use of electronic health record (EHR)-embedded child abuse clinical decision support (CA-CDS) may help decrease morbidity from child maltreatment. We previously reported on the development of CA-CDS in Epic and Allscripts. The objective of this study was to implement CA-CDS into Epic and Allscripts and determine its effects on identification, evaluation, and reporting of suspected child maltreatment.

**Materials and Methods:**

After a preimplementation period, CA-CDS was implemented at University of Wisconsin (Epic) and Northwell Health (Allscripts). Providers were surveyed before the go-live and 4 months later. Outcomes included the proportion of children who triggered the CA-CDS system, had a positive Child Abuse Screen (CAS) and/or were reported to Child Protective Services (CPS).

**Results:**

At University of Wisconsin (UW), 3.5% of children in the implementation period triggered the system. The CAS was positive in 1.8% of children. The proportion of children reported to CPS increased from 0.6% to 0.9%. There was rapid uptake of the abuse order set.

At Northwell Health (NW), 1.9% of children in the implementation period triggered the system. The CAS was positive in 1% of children. The child abuse order set was rarely used. Preimplementation, providers at both sites were similar in desire to have CA-CDS system and perception of CDS in general. After implementation, UW providers had a positive perception of the CA-CDS system, while NW providers had a negative perception.

**Discussion:**

CA-CDS was able to be implemented in 2 different EHRs with differing effects on clinical care and provider feedback. At UW, the site with higher uptake of the CA-CDS system, the proportion of children who triggered the system and the rate of positive CAS was similar to previous studies and there was an increase in the proportion of cases of suspected abuse identified as measured by reports to CPS. Our data demonstrate how local environment, end-users’ opinions, and limitations in the EHR platform can impact the success of implementation.

**Conclusions:**

When disseminating CA-CDS into different hospital systems and different EHRs, it is critical to recognize how limitations in the functionality of the EHR can impact the success of implementation. The importance of collecting, interpreting, and responding to provider feedback is of critical importance particularly with CDS related to child maltreatment.

## INTRODUCTION

Child maltreatment is a leading cause of morbidity and mortality in children^1^^,^^2^ Data from numerous studies over several decades demonstrate that many children who die or nearly die from abuse have been previously evaluated for an injury which was not recognized as abusive.^3–10^ Many of these missed opportunities occur in emergency department (ED) settings. Despite ongoing education of ED providers, missed abuse continues to be challenging and the proportion of abused children with prior missed opportunities to diagnose abuse has been stable.^10^

Using electronic health record (EHR)-embedded child abuse clinical decision support (CA-CDS) may help decrease missed abuse by assisting providers to identify, evaluate, and report suspected child maltreatment. We previously reported on the development and implementation of CA-CDS embedded in Cerner at the University of Pittsburgh Medical Center.^11–15^ This CA-CDS system has been part of clinical practice at our level I pediatric trauma center since late 2015 and at the general EDs since early 2017. More recently, we reported on the process of developing CA-CDS in 2 other EHRs—Epic and Allscripts—at Northwell Health (NW) and University of Wisconsin (UW).^16^ Each system is triggered through a combination of a universal Child Abuse Screen (CAS) and chief complaints which are associated with child maltreatment. The UW system also uses ICD-10 codes as a trigger. Once a patient triggers the system, the provider is alerted to the concern, led to a child physical abuse order set, and receives assistance making a report of suspected maltreatment. In this article, we describe the implementation of CA-CDS in Epic and Allscripts.

## MATERIALS AND METHODS

This study was approved by the Institutional Review Boards at UW which uses Epic (Epic, Verona, WI, USA), and NW which uses Allscripts (Sunrise Clinical Manager, Allscripts, Chicago, IL, USA).

### Setting

There were 2 participating EDs at UW: UW Health BerbeeWalsh ED supporting the American Family Children’s Hospital (pediatric ED as part of a general academic ED with pediatric and adult level I trauma/burn center), and East Madison Hospital (general community ED). There were 4 participating EDs at NW: Cohen Children’s Medical Center (pediatric level I trauma center), North Shore University Hospital (adult level I trauma center), Long Island Jewish Forest Hills (general community ED), and Staten Island University Hospital (pediatric level II/adult level I trauma center). For both healthcare systems, the word “provider” refers to physicians and/or advanced practice providers.

### Subjects

Children <10 years old evaluated at UW and children <13 years old at NW were eligible to have a CAS completed and were included in data collection. The triggers, alerts, and orders sets in each system have been described previously.^16^

### Pre- and postsurveys

A 42-question survey was sent by email to all providers at each site prior to the CA-CDS implementation (Supplementary Appendix SA). The surveys assessed providers’ level of confidence in recognizing, evaluating, and reporting physical abuse, their perceptions of whether they and their colleagues over- or under-evaluate for abuse and their interactions and opinions about the EHR and EHR-embedded CDS, in general. A 17-question follow-up survey was sent ∼4 months after CA-CDS implementation to collect information about the providers’ experience with CA-CDS (Supplementary Appendix SB).

### Data collection

#### Subject-specific data

The following data were downloaded directly from the EHR for all children evaluated at each hospital system during both the preimplementation and implementation periods: demographics, visit details including visit date, chief complaint, whether the child was transferred from another facility, ICD-10 codes, whether there was a social work consult, results of the skeletal survey, abdominal computed tomography (CT), plain films and neuroimaging, and lab tests which are part of the physical abuse order sets. CA-CDS-specific information was also collected and included the result of the CAS, whether the patient triggered the CA-CDS system and if so, what the initial trigger was, whether a report was made to Child Protective Services (CPS) and whether a physical abuse order set was used.

Race was assigned by the registrar at each site using a drop-down menu and analyzed as a dichotomous variable (White vs not White). Initial trigger was defined as the trigger which resulted in the first notification to the ED provider. Possible initial triggers included (1) chief complaint, (2) positive CAS, (3) orders, or (4) ICD-10 (UW only). In the preimplementation dataset, whether the patient triggered was hypothetical and based on whether the patient would have triggered had the system been active. There was no CAS in this period.

#### Data collection periods

At UW, the preimplementation period was June 1, 2018 to March 31, 2019 (10 months); implementation was April 1, 2019 to March 31, 2020 (12 months). At NW, the preimplementation period was May 1, 2018 to February 28, 2019 (10 months); implementation was June 18, 2019 to April 15, 2020 (10 months). There was a 3-month period at NW from March 26, 2019 to June 17, 2019 during which coding errors in the clinical decision support prevented the providers from consistently receiving alerts. Data from this period were not included in the analysis since this period was neither clearly preimplementation nor implementation since some, but not all, of the alerts and acknowledgments were functioning.

### Primary outcome measures

The primary outcome measures were the proportion of children with a positive CAS (defined as any question in the CAS being positive), proportion of children who triggered the CA-CDS system, proportion of children who were reported to CPS and proportion of providers who complied with the American Academy of Pediatrics (AAP) recommendation for evaluation of children <2 years old with specific clinical scenarios. The clinical scenarios were (1) bruise in a <6-month-old, (2) fracture in a <12-month-old, (3) intracranial hemorrhage in a <12-month-old, and (4) report to CPS for concerns of physical abuse in a <2-year-old with an injury. Patients with the first 3 clinical scenarios were identified by combining age with ICD-10 codes. For the fourth scenario, a report to CPS was defined by the completion of a child abuse-specific form in the EHR. UW had this form prior to CA-CDS implementation; NW developed it as part of integration. These clinical scenarios were chosen because the AAP has specific guidelines for which radiologic and lab testing should be completed.^17–19^ They represent only a subset of the situations in which it would be reasonable for a provider to evaluate for abuse.

Compliance with AAP guidelines was determined using data downloaded from the EHR except when a child was transferred from another hospital in which case the site investigators queried the EHR directly to determine what testing was done prior to transfer. Each evaluation for physical abuse was assessed as “fully compliant,” “partially compliant,” “not compliant,” or “met clinical scenario but clinical judgment made evaluation unnecessary.”

“Fully compliant” was defined as completing all the AAP recommended testing, “partially compliant” was defined as completing a subset of the recommended tests and “not compliant” was defined as performing no abuse evaluation. “Met scenario but clinical judgment made evaluation unnecessary” was defined as in our previous studies^11^^,^^14^ and used in the following circumstances: injury occurred in a public place and/or witnessed by a disinterested adult, infant was developmentally able to cruise and had a toddler’s fracture or the patient had a pre-existing diagnosis (eg, hemophilia) based on ICD-coding which would clearly explain the injury; subjects in this group were included in the full compliant category since the provider correctly assessed that an abuse evaluation was not needed. All cases designated as “not compliant” were reviewed by the site team to determine if any of these circumstances were applicable.

### Statistical analysis

Descriptive statistics were used to examine demographic and compliance variables during both time periods in each hospital system. SPSS 25.0 (Chicago, IL, USA) was used for all analyses. A *P *<* *.05 was considered significant.

## RESULTS

### Patient demographics and overall CA-CDS characteristics during preimplementation and implementation periods

#### University of Wisconsin

During the 10-month preimplementation period, 8159 children <10 years old were evaluated; 11 397 children were evaluated during the 12-month implementation period. In both periods, 87% of subjects were seen at the American Family Children’s Hospital ED. There was a child abuse pediatrician during the preimplementation period; a physician assistant and general pediatrician provided consultation during implementation. Patient demographics (Table 1) did not differ between the 2 periods. The mean (SD) age of children who triggered the CA-CDS system was younger than those who did not (20.1 [24.2] vs 45.0 [33.8] months [*P *<* *.00]) and did not differ by period. Children with public insurance were more likely to trigger than those with private insurance (*P *<* *.00) in both periods. There was no difference in triggering by race (*P *=* *.30), but there was a strong relationship between insurance and race with a greater proportion of non-White vs White children having Medicaid (68% vs 30%, *P *<* *.00) in both periods.

**Table 1.** Overall demographics of the University of Wisconsin and Northwell Health sites

	University of Wisconsin	Northwell Health
Mean (SD) age in months	44.3 (34.0)	58.4 (45.5)
Race (% non-White)	26%	67%
Gender (% male)	56%	55%
Insurance (% public)	38%	32%

#### Northwell Health

During the 10-month preimplementation, 46 884 children <13 years old were evaluated; 47 953 were evaluated during the 10-month implementation. Sixty-two percent were seen at Cohen Children’s Medical Center, 25% at Staten Island University Hospital, 7% at Long Island Jewish Forest Hills, and 6% at North Shore University Hospital. There was a child abuse pediatrician at Staten Island University Hospital but not at any of the other hospitals during both preimplementation and implementation. Patient demographics (Table 1) did not differ between periods. The mean age of children who triggered was younger than those who did not (28.5 [40.4] vs 58.7 [45.4] months, *P *<* *.00). Children with public insurance were more likely to trigger both periods (*P *<* *.00). There was no relationship between the likelihood of triggering and race (*P *=* *.7), but a strong relationship between insurance and race with a greater proportion of non-White vs White children having Medicaid (42% vs 28%, *P *<* *.00).

### Preimplementation surveys

Preimplementation surveys were completed by 39 providers - 19 at UW (28% response rate) and 20 at NW (22% response rate). Responses from UW and NW providers were similar. At both sites ∼50% of providers felt they under-evaluated for child abuse and ∼50% felt that their colleagues did. Only one respondent felt their colleagues over-evaluated for abuse. When asked how they make decisions about when to evaluate for abuse, almost 70% of providers “remember what to do based on my experience/training,” 40% use an on-line resource, and 40% discuss with a colleague. Almost all the UW providers also selected that they page the child abuse pediatrician When asked what proportion of skeletal surveys they felt should be positive (demonstrate an unexpected fracture) to justify the risk associated with performing them, providers at both sites responded that about 1 of 50 (∼2%) should be positive. Just over 70% of providers at both sites were looking forward to having CA-CDS.

When asked about the EHR in general, ∼40% of providers at both sites “somewhat agreed” that they were satisfied with the EHR; over 30% strongly disagreed, disagreed, or somewhat disagreed. There were no statistically significant differences in the responses to any of the EHR-related questions between sites.

### CA-CDS system: preimplementation

#### University of Wisconsin

Overall, 2.4% of children during the preimplementation period triggered the CA-CDS system (Table 2). The proportion of children reported to CPS was 0.6% (49/8159). A total of 0.6% of all children met one of the clinical scenario and compliance with AAP recommendations was 80% (Table 3).

**Table 2.** The proportion of children who triggered the child abuse alert system and the reasons for triggering at University of Wisconsin and Northwell Health during the preimplementation and implementation periods

	Preimplementation	Implementation
Any trigger		
UW	2.4% (199/8159)	3.5% (395/11 397)
NW	1.1% (514/46 885)	1.7% (821/47 941)
Child abuse screen		
UW	Not applicable	1.8% (210/11 397)
NW	Not applicable	1.0% (470/47 943)
Chief complaint		
UW	1.8% (152/8159)	1.5% (172/11 397)
NW	0.7% (335/46 885)^a^	0.7% (337/47 953)
Order		
UW	0.9% (77/8159)	0.7% (82/11 397)
NW	0.4% (194/46 885)	0.4% (185/47 953)
Discharge/ICD-10 code		
UW	0.9% (74/8159)	0.8% (94/11 397)
NW	Not applicable	Not applicable

a Based on extrapolation since the chief complaints used to trigger did not yet exist.

**Table 3.** Compliance with American Academy of Pediatrics recommendations for evaluation of physical abuse during the preimplementation and implementation periods at University of Wisconsin and Northwell Health

	Preimplementation	Implementation	*P*-value
Number of cases meeting criteria			
UW	52	65	Not applicable
NW	124	111	Not applicable
Non-compliant			
UW	10% (5/52)	14% (14/65)	.15
NW	44% (55/124)	31% (35/111)	.18
Partially compliant			
UW	10% (5/52)	11% (7/65)	.88
NW	23% (28/124)	20% (22/111)	.68
Fully compliant or met clinical scenario but clinical judgment made evaluation unnecessary			
UW	80% (42/52)^a^	75% (49/65)	.82
NW	33% (41/124)^b^	50% (55/111)	.09

a Three children in the preimplementation and 5 in the implementation periods at UW were appropriately assessed as “met clinical scenario but clinical judgment made evaluation unnecessary.” These were included in the full compliance group since the provider made a correct assessment and did not over-evaluate for abuse.

b Six children in the preimplementation and 0 in implementation at NW were appropriately assessed as “met clinical scenario but clinical judgment made evaluation unnecessary.” These were included in the full compliance group since the provider made a correct assessment and did not over-evaluate for abuse.

#### Northwell Health

Overall, 1.1% of children during the preimplementation period triggered the CA-CDS system (Table 2). The EHR-integrated CPS reporting form was implemented at the time of the CA-CDS implementation. Prior to that, documentation of CPS reporting was done by free text in social work notes which were not part of the EHR. As a result, the proportion of children reported to CPS preimplementation could not be calculated. A total of 0.3% of children preimplementation met a clinical scenario and compliance with AAP recommendations was 33% (Table 3).

### CA-CDS system: implementation characteristics

#### University of Wisconsin

Overall, 3.5% of children in the implementation period triggered the CA-CDS system (Table 2). The CAS was completed in 80.2% children <10 years old with no difference between hospitals. Overall, 1.8% of the CAS were positive. Of children with a positive CAS, question 1 (delay in seeking care) was positive in 0.5% (*n* = 60), question 2 (history not consistent with injury) in 0.5% (*n* = 53), question 3 (abnormal physical exam finding) in 0.5% (*n* = 49), question 4 (concern for neglect) in 0.6% (*n* = 72), and question 5 (open response) in 0.6% (*n* = 67). Examples of open responses are in Supplementary Appendix SC.

There was an increase in the proportion of children reported to CPS to 0.9% (101/11397) from 0.6% (49/8159) (*P *=* *.03). The increase was across all types of abuse. Seventeen percent of children who triggered the CA-CDS system were reported to CPS compared with 0.26% of those who did not trigger (*P *<* *.00). Children with a positive CAS were more likely to be reported than those with a negative CAS (20% [21/107] vs 0.4% [28/7006], *P *<* *.00).

The same proportion of all children met one of the clinical scenario and compliance with AAP recommendations was unchanged from the preimplementation (Table 3). Uptake of the physical abuse order set was rapid; over 50% of the children in the implementation period with a clinical scenario had an order set used.

#### Northwell Health

Overall, 1.9% of children in the implementation period triggered the CA-CDS system (Table 3). During implementation, the rate of reports to CPS based on the new CPS reporting form was 0.3% (101/11397); 8.8% (72/822) of children who triggered the CA-CDS were reported.

The CAS was completed in 70% of children; 1.0% were positive. Of children with a positive CAS, question 1 (delay in seeking care) was positive in 0.3% (*n* = 111), question 2 (history not consistent with injury) in 0.2% (*n* = 83), question 3 (abnormal physical exam finding) in 0.5% (*n* = 164), question 4 (concern for neglect) in 0.3% (*n* = 125), and question 5 (open response) in 0.3% (*n* = 135). Examples of open responses are in Supplementary Appendix SC.

Overall, 0.2% of children met one of the clinical scenario and compliance with AAP recommendations; compliance with AAP recommendations was 50% during implementation (Table 3). The order set was only used 23 times during implementation; compliance was 96% (22/23) when it was used.

### Postimplementation surveys

Postimplementation surveys were completed by 89 providers - 27 at UW (29% response rate) and 62 at NW (67% response rate). UW providers were more likely than NW providers to agree/strongly agree that the CA-CDS system “increases awareness of the potential risk for child abuse” (90% vs 27%, *P* < .00) and to agree/strongly agree that the “child abuse alert is helpful to my clinical decision making” (65% vs 31%, *P *=* *.08). Desire to continue using CA-CDS was much stronger at UW compared with NW (81% vs 3%, *P *<* *.00).

Provider free text comments from UW were generally positive, with providers noting the CA-CDS system is “a helpful reminder” and “extremely helpful for standardizing care.” There were only a few comments from NW providers. One read: “I feel like I don’t notice the alert until I am completing the note when something pops up…It would be more useful if this popped up as soon as I opened the note for the first time, or….before I examine the patient so I can think to look for other more specific signs of abuse.” The technical limitation of Allscripts’ EHR to alert the provider until he/she started a patient note was a recognized shortcoming and had been discussed by the implementation team at length prior to implementation.

Providers at the 2 sites had very different opinions of the alert and order sets; 76% of UW providers, but only 8% of the NW providers “agree/strongly agree” that the alert and order sets are useful (*P *<* *.00). More UW providers agree/strongly agree that the order sets fit well into their clinical workflow (57% vs 11%, *P* = <.00). There was a marked difference in whether providers agreed with the AAP recommendations included in the abuse order set; 76% (16/21) of UW providers agreed/strongly agreed with the suggested workup, while 71% (44/62) of NW providers disagreed/strongly disagreed with it.

## DISCUSSION

This is the first study, to our knowledge, to disseminate EHR-embedded CA-CDS into different EHRs in different hospital systems. Our data demonstrate the importance of the local environment and end-user feedback in the implementation process and how limitations of the EHR platform impact the success of implementation. While the impact of the CA-CDS integration on the primary outcomes was not as robust as one might have liked or anticipated based on previous data, the lessons learned from the challenges faced during the integration can influence future research.

The rate of triggering of the CA-CDS system was lower at NW for both preimplementation and after implementation. While NW was unable to use ICD-10 codes as triggers, this was not the primary reason for the lower triggering rate since it was not as common as other triggers and was frequently a secondary trigger (eg, patient had already triggered for another reason). But within each of the other trigger categories—CAS, chief complaint, and orders—a smaller proportion of patients triggered at NW. The proportion of positive CAS was almost 2-fold higher at UW (1.8% vs 1.0%). While it is not possible to know the “right” rate of positive CAS, several studies in the US and abroad have demonstrated a positive rate in the 1.8%–2.2% range.^13^^,^^20^^,^^21^ Given that the annual rate of substantiated maltreatment—maltreatment which is determined to meet a given state’s definition after a complete investigation—is ∼1% among children <18 years of age in the United States,^1^ one would expect that a *screening* tool such as the CAS would result in a positivity rate >1%.

The difference in the proportion of chief complaint triggering between the 2 sites was surprising given how many more chief complaints could trigger at NW.^16^ Due to technical limitations, Allscripts EHR was not able to link patient age and a chief complaint; as a result, new chief complaints which included both age and chief complaint (eg, “bruise <1 yr.”) were developed and nurses were trained to use them. As a result, it is likely that there were NW patients who should have triggered for a chief complaint but did not because of the way the chief complaint was entered (eg, selection of chief complaint of “bruise” in a <1 year old. vs selection of “bruise <1 yr.”). The inability of Allscripts to trigger based on free text chief complaints was one of several EHR-specific factors which likely limited the success of the CA-CDS implementation.

The difference in triggering based on orders is perhaps the most striking and unexpected. At UW, ordering of a skeletal survey in a child <5 years old outside of the abuse order set was the only order-based trigger. At NW, ordering of a skeletal survey in a child <2 years old outside the order set, as well as any X-ray order (other than chest or abdomen) in a <1 year old triggered; one would expect many more triggers because of the use of plain films as a trigger. While the proportion of children undergoing skeletal surveys was much lower at NW than UW, one would have anticipated that the use of plain films would more than make up for the lack of skeletal surveys. After evaluation of multiple hypotheses, including the possibility that there was an error in the EHR report, we believe that there were other orders for X-rays which were not coded into the CDS system so that the orders were not correctly triggering.

Just as it is difficult to know the “right” rate of triggering, it is difficult to assess the “right” rate of CPS reporting. According to the National Child Abuse and Neglect Data System, there are ∼3 million reports of abuse annually involving 6 million children,^1^ suggesting that ∼8% of the 73 million children in the United States are reported to CPS annually. While the rate of reporting for any given ED visit is certainly lower than 8%, it is also certainly much higher than 0.02% which is the unadjusted daily rate of reporting (8/365). Children who present to the ED often present for a concerning event or with ongoing concerns which are more likely to be recognized and reported by medical providers because of their experience and expertise. It is, therefore, difficult to know whether the increase in the reporting rate at UW after implementation of the CA-CDS system to 0.9% is a higher or lower proportion than expected.

The similarity in providers between sites preimplementation and the divergence after implementation suggests that it was the functioning and interface of the CA-CDS, rather than a difference in the providers themselves, that was at least partially responsible for our findings. The low triggering rate—which meant that many high-risk patients did not alert—the lack of interruptive alerts at NW and the inability to directly link to the order set from an alert likely contributed to the low compliance with AAP recommendations and the negative feedback. A recently published consensus statement on CA-CDS^15^ specifically notes that these characteristics—high sensitivity, use of interruptive alerts and direct linkage to the order set—are critical components of a CA-CDS system. The lack of agreement of the NW providers with the AAP recommendations for evaluation of physical abuse seen on the postimplementation survey was unexpected. Prior to implementation, the research team as well as the site PI at NW had multiple meetings and presentations with the NW providers about the CA-CDS system, including the CAS, the alerts/acknowledgments, and order sets. While providers gave feedback about virtually every aspect of the CA-CDS system, no providers expressed concern about the order sets prior to the implementation. In the future, providers will specifically be asked on the preimplementation surveys to review and comment on whether they agree with the AAP recommendations for evaluation of physical abuse in young children.

The technical limitations of the CA-CDS system at NW do not entirely explain the pre- to postimplementation divergence in the survey results. The high level of disagreement of NW providers with the AAP recommendations was unexpected and had not been expressed in multiple educational sessions or face-to-face discussions. While the same proportion of providers at both sites felt that they under-evaluated for abuse, when offered evidence-based order sets to assist in increasing and improving evaluation for abuse, the NW providers did not use them. In hindsight, this is, at least in part, due to their disagreement with the recommendations in the order sets but there are likely additional factors. Preliminary discussions have yielded additional critical information. First, NW providers do not routinely use order sets in general. Second, and specific to child abuse, there also appears to be a lack of trust in the CPS system which may affect the willingness of providers to evaluate for and report suspected abuse. The lack of a child abuse pediatrician during the preimplementation and most of the implementation period, as well as the system functioning limitations, most certainly exacerbated these issues. It is also important to consider that the NW system was significantly larger than the UW system both in terms of patient volume (90 000 vs 19 000 visits during preimplementation and implementation) and number of sites (4 vs 2). The larger number of patients and sites means there are more nurses and providers to educate and likely more variability in practice. A final limitation is the low response rates to the surveys; we do not know if the respondents were a representative sample of providers at each site.

## CONCLUSIONS

Our data demonstrate that while it is possible to disseminate CA-CDS into different hospital systems and different EHRs, it is critical to recognize how limitations in the functionality of the EHR can impact the success of implementation. In addition, the importance of collecting, interpreting, and responding to provider feedback is of critical importance particularly with CDS related to child maltreatment. Multiple studies over several decades demonstrate that medical providers’ decision-making related to child maltreatment cases can be influenced by factors including implicit bias, previous experiences with the CPS system, and negative media reports about the CPS system.^22–25^ These factors may have influenced the effectiveness of the CA-CDS implementation and particularly the specific primary outcome measures which were selected for this project. Future research will focus on how these factors may influence implementation effectiveness and whether behavioral economics might be able to address these potential barriers to CA-CDS implementation.

## Supplementary Material

ooad022_Supplementary_Data

## Data Availability

The data underlying this article will be shared upon reasonable request to the corresponding author.
