# Supplementary material for: Disseminating child abuse clinical decision support among commercial electronic health records: Effects on clinical practice
Source: JAMIA Open. 2023 Apr 13;6(2):ooad022. doi: 10.1093/jamiaopen/ooad022 (PMC10101685; doi:10.1093/jamiaopen/ooad022)
Supplement: ooad022_Supplementary_Data [file ooad022_supplementary_data.zip › Appendix B- provider post-survey.docx]

APPENDIX B: Provider Post-Implementation Survey

Please answer the following questions regarding the child abuse clinical decision support system (CA-CDS) that was implemented in the Emergency Department. The CA-CDS includes best practice ALERTS based on chief complaint, nurse child abuse screen and diagnosis with ORDER SETS for bruise, burn, fracture, skull fracture, intracranial injury, other injuries and neglect.

**1.     On average, how often per shift do you receive an ALERT from the CA-CDS?** (Give your best estimate and it is okay to use a decimal) _____

Please use the scale below to rate you agreement with the following statements about the CA-CDS

**2.**     **The CA-CDS increases my awareness of the potential risk for child abuse.**

Strongly Disagree-1----Disagree-2----Neutral-3----Agree-4----Strongly Agree-5

**3.**     **The alert I receive from the CA-CDS is clearly worded.**

Strongly Disagree-1----Disagree-2----Neutral-3----Agree-4----Strongly Agree-5

**4.**     **When I receive a CA-CDS alert it is clear why it triggered.**

Strongly Disagree-1----Disagree-2----Neutral-3----Agree-4----Strongly Agree-5

**5.**     **When I receive a CA-CDS alert I know how to silence it if so desired.**

Strongly Disagree-1----Disagree-2----Neutral-3----Agree-4----Strongly Agree-5

**6.**     **When I receive a CA-CDS alert I know which orderset to choose.**

Strongly Disagree-1----Disagree-2----Neutral-3----Agree-4----Strongly Agree-5

**7.**     **The alert and associated order sets are a useful tool at the point of care (**i.e. it is appropriate/improves quality of patient care**)**

 Strongly Disagree-1----Disagree-2----Neutral-3----Agree-4----Strongly Agree-5

**8. Using the child abuse order sets fits well in my clinical workflow*.***

 Strongly Disagree-1----Disagree-2----Neutral-3----Agree-4----Strongly Agree-5

**9. The child abuse alert is helpful to my clinical decision making*.***

Strongly Disagree-1----Disagree-2----Neutral-3----Agree-4----Strongly Agree-5

**10.**    **The child abuse alert and associated order sets limit my ability to make independent decisions*.***

 Strongly Disagree-1----Disagree-2----Neutral-3----Agree-4----Strongly Agree-5

**11.**   **The child abuse order sets saves time when evaluating patients*.***

Strongly Disagree-1----Disagree-2----Neutral-3----Agree-4----Strongly Agree-5

**12.**  **I want to continue using the CA-CDS.**

Strongly Disagree-1----Disagree-2----Neutral-3----Agree-4----Strongly Agree-5

12a. If you disagree please explain.

**13. I agree with the suggested workup in the order sets (**bruise, burn, fracture, skull fracture, intracranial injury, other injuries and neglect).

Strongly Disagree-1----Disagree-2----Neutral-3----Agree-4----Strongly Agree-5

13a. If you disagree please explain.

**Please rate the following questions on a scale of 0 – 10 with 0 being least useful and 10 being most useful**

**14.**  **How useful is the CA-CDS including alerts and order sets?**

0----1----2----3----4----5---6---7----8----9----10

**15. How useful is getting an alert to help you consider physical abuse or neglect?**

0----1----2----3----4----5---6---7----8----9----10

**16. How useful are the abuse order sets (**bruise, burn, fracture, skull fracture, intracranial injury, other injuries and neglect)?

0----1----2----3----4----5---6---7----8----9----10

**17. Please provide any other comments that you would like to share with us about the CA-CDS?** [Free text box]
