# Supplementary material for: Disseminating child abuse clinical decision support among commercial electronic health records: Effects on clinical practice
Source: JAMIA Open. 2023 Apr 13;6(2):ooad022. doi: 10.1093/jamiaopen/ooad022 (PMC10101685; doi:10.1093/jamiaopen/ooad022)
Supplement: ooad022_Supplementary_Data [file ooad022_supplementary_data.zip › Appendix C- free text comments.docx]

| “grandmother swearing at child and fake slapping the child on the face” |
| --- |
| “co-sleeping, unwitnessed fall with unknown downtown, mother seems detached, continues to state how exhausted she is” |
| “pt generally dirty, cuts on bottom of feet and bruising to heel” |
| “care delayed x 18 hours” |
| “fall x 2 days with change in behavior and bulging fontanel…mother advised to bring to ED and did not” |

APPENDIX C: Comments provided for the free-text question of the Child Abuse Screen by site

Sample of comments provided for the free-text question of the child abuse screen at

University of Wisconsin

| “father called xxxx because he states mother didn't come home last night and then locked his son out of the house today” |
| --- |
| “Pt. states father pushed her because he was mad” |
| “Patient found alone wondering streets with no coat- mom states "she can undo any lock"” |
| “mother verbalizes being abused by father of child” |
| “pt endorses father hitting her in the stomach while wearing boxing gloves” |
| “Small abrasion noted where pt. claims father "punched him."” |
| “Pt states was hit and spit by father . Pt states hes afraid of his uncle who he lives with” |
| “swelling of L eye from w here mom hit pt with closed hand” |
| “pt was physically hit by leather belt by the father” |

Sample of comments provided for the free-text question of the child abuse screen at Northwell
