# Supplementary material for: Disseminating child abuse clinical decision support among commercial electronic health records: Effects on clinical practice
Source: JAMIA Open. 2023 Apr 13;6(2):ooad022. doi: 10.1093/jamiaopen/ooad022 (PMC10101685; doi:10.1093/jamiaopen/ooad022)
Supplement: ooad022_Supplementary_Data [file ooad022_supplementary_data.zip › Appendix A- provider pre-survey.docx]

APPENDIX A: Provider Pre-implementation Survey

Approximately how many cases of suspected child abuse (physical, sexual or other) did you evaluate in the past year? ______

Overall, do you feel that *you*

- Over-evaluate for child abuse
- Under-evaluate for child abuse
- Evaluate in an appropriate number of cases

Overall, do you feel that *other physicians in your ED*

- Over-evaluate for child abuse
- Under-evaluate for child abuse
- Evaluate in an appropriate number of cases

How do you decide what tests to do for a child in whom you suspect physical abuse? (Select all that apply)

- I remember what to do based on my experience/training
- I use an-online resources
- I page the child abuse pediatrician on call.
- I discuss with a colleague
- Other, please describe ________________________________________________

I am confident in my ability to recognize when a child might be a victim of **physical abuse**

(0-10 scale with 0=not at all confident and 10=extremely confident)

0----1----2----3----4----5---6---7----8----9----10

I am confident in my ability to evaluate children with suspected physical abuse.

(0-10 scale with 0=not at all confident and 10=extremely confident)

0----1----2----3----4----5---6---7----8----9----10

I am confident in my ability to document a diagnosis of suspected or definite physical abuse in the EHR.

(0-10 scale with 0=not at all confident and 10=extremely confident)

0----1----2----3----4----5---6---7----8----9----10

I am confident in my ability to recognize when a child might be a victim of **sexual abuse**.

(0-10 scale with 0=not at all confident and 10=extremely confident)

0----1----2----3----4----5---6---7----8----9----10

I am confident in my ability to evaluate children with suspected sexual abuse.

(0-10 scale with 0=not at all confident and 10=extremely confident)

0----1----2----3----4----5---6---7----8----9----10

I am confident in my ability to recognize when a child might be a victim of **neglect**.

(0-10 scale with 0=not at all confident and 10=extremely confident)

0----1----2----3----4----5---6---7----8----9----10

I am confident in my ability to evaluate children with suspected neglect.

(0-10 scale with 0=not at all confident and 10=extremely confident)

0----1----2----3----4----5---6---7----8----9----10

I am confident in my ability to know what information should be included in a report to Child Protective Services.

(0-10 scale with 0=not at all confident and 10=extremely confident)

0----1----2----3----4----5---6---7----8----9----10

Which of the following issues do you consider when deciding whether to order a skeletal survey to evaluate for suspected physical abuse in a child under 2 years of age? (select all which apply)

- Concern about radiation exposure
- Concern that I am accusing parents of child abuse
- Concern that the yield is too low to justify it
- Concern that it will greatly extend the ED length of stay
- None of the above
- Other, please describe ­­­­­­­­­­­­­­­________________________________________

Approximately what proportion of skeletal surveys do you feel should be positive (defined as identifying an unexpected fracture) to justify the risk associated with them?

- 1 of 5 (20%)
- 1 of 10 (10%)
- 1 of 20 (5%)
- 1 of 50 (2%)
- Other _________________________________

Are you looking forward to having a child abuse clinical decision support system (CA-CDSS) to help with identifying, evaluating and reporting child abuse?

- Yes
- Not sure
- No

If you answered ‘not sure’ or ‘no’ to the question above, can you tell us why? (select all that apply)

- I am concerned about too many pop-ups/over-triggers ('alert fatigue')
- I am already comfortable in my ability to recognize and evaluate abuse and don’t feel I need more guidance
- I feel that we already evaluate too many children for child abuse
- Concern that the tool might not be accurate
- Other, please describe ________________________________________________

All of the following questions pertain to your experience with the electronic health record (EHR) in its entirety, including any clinical decision support (CDS) you interact with (alerts, pop-ups, decision

aids). Please answer each question with: 1=Strongly disagree, 2=Disagree, 3=Somewhat disagree, 4=Neither disagree nor agree, 5=Somewhat agree, 6=Agree or 7=Strongly agree.

I am satisfied with the quality of information available in the EHR

1----------------- 2 ----------------- 3 -------------------- 4 --------------------- 5 ----------------- 6 ----------------- 7

| Strongly disagree | Disagree | Somewhat disagree | Neither disagree nor agree | Somewhat agree | Agree | Strongly agree |
| --- | --- | --- | --- | --- | --- | --- |

EHR use is compatible with all aspects of my work

1----------------- 2 ----------------- 3 -------------------- 4 --------------------- 5 ----------------- 6 ----------------- 7

| Strongly disagree | Disagree | Somewhat disagree | Neither disagree nor agree | Somewhat agree | Agree | Strongly agree |
| --- | --- | --- | --- | --- | --- | --- |

I am satisfied with the reliability of the EHR

1----------------- 2 ----------------- 3 -------------------- 4 --------------------- 5 ----------------- 6 ----------------- 7

| Strongly disagree | Disagree | Somewhat disagree | Neither disagree nor agree | Somewhat agree | Agree | Strongly agree |
| --- | --- | --- | --- | --- | --- | --- |

The EHR is always available when I need it

1----------------- 2 ----------------- 3 -------------------- 4 --------------------- 5 ----------------- 6 ----------------- 7

| Strongly disagree | Disagree | Somewhat disagree | Neither disagree nor agree | Somewhat agree | Agree | Strongly agree |
| --- | --- | --- | --- | --- | --- | --- |

I am satisfied with the speed of the EHR

1----------------- 2 ----------------- 3 -------------------- 4 --------------------- 5 ----------------- 6 ----------------- 7

| Strongly disagree | Disagree | Somewhat disagree | Neither disagree nor agree | Somewhat agree | Agree | Strongly agree |
| --- | --- | --- | --- | --- | --- | --- |

I am satisfied with the confidentiality offered by the EHR

1----------------- 2 ----------------- 3 -------------------- 4 --------------------- 5 ----------------- 6 ----------------- 7

| Strongly disagree | Disagree | Somewhat disagree | Neither disagree nor agree | Somewhat agree | Agree | Strongly agree |
| --- | --- | --- | --- | --- | --- | --- |

The EHR is easy for me to use

1----------------- 2 ----------------- 3 -------------------- 4 --------------------- 5 ----------------- 6 ----------------- 7

| Strongly disagree | Disagree | Somewhat disagree | Neither disagree nor agree | Somewhat agree | Agree | Strongly agree |
| --- | --- | --- | --- | --- | --- | --- |

The use of the EHR improves my efficiency in my professional work practice

1----------------- 2 ----------------- 3 -------------------- 4 --------------------- 5 ----------------- 6 ----------------- 7

| Strongly disagree | Disagree | Somewhat disagree | Neither disagree nor agree | Somewhat agree | Agree | Strongly agree |
| --- | --- | --- | --- | --- | --- | --- |

The use of the EHR improves my decision making

1----------------- 2 ----------------- 3 -------------------- 4 --------------------- 5 ----------------- 6 ----------------- 7

| Strongly disagree | Disagree | Somewhat disagree | Neither disagree nor agree | Somewhat agree | Agree | Strongly agree |
| --- | --- | --- | --- | --- | --- | --- |

The EHR is generally useful in my professional practice

1----------------- 2 ----------------- 3 -------------------- 4 --------------------- 5 ----------------- 6 ----------------- 7

| Strongly disagree | Disagree | Somewhat disagree | Neither disagree nor agree | Somewhat agree | Agree | Strongly agree |
| --- | --- | --- | --- | --- | --- | --- |

I am satisfied with the EHR training

1----------------- 2 ----------------- 3 -------------------- 4 --------------------- 5 ----------------- 6 ----------------- 7

| Strongly disagree | Disagree | Somewhat disagree | Neither disagree nor agree | Somewhat agree | Agree | Strongly agree |
| --- | --- | --- | --- | --- | --- | --- |

I am satisfied with the quality of the available computer material available in the units

1----------------- 2 ----------------- 3 -------------------- 4 --------------------- 5 ----------------- 6 ----------------- 7

| Strongly disagree | Disagree | Somewhat disagree | Neither disagree nor agree | Somewhat agree | Agree | Strongly agree |
| --- | --- | --- | --- | --- | --- | --- |

I am satisfied with the EHR support services

1----------------- 2 ----------------- 3 -------------------- 4 --------------------- 5 ----------------- 6 ----------------- 7

| Strongly disagree | Disagree | Somewhat disagree | Neither disagree nor agree | Somewhat agree | Agree | Strongly agree |
| --- | --- | --- | --- | --- | --- | --- |

The hospital hierarchy prompts me to use the EHR

1----------------- 2 ----------------- 3 -------------------- 4 --------------------- 5 ----------------- 6 ----------------- 7

| Strongly disagree | Disagree | Somewhat disagree | Neither disagree nor agree | Somewhat agree | Agree | Strongly agree |
| --- | --- | --- | --- | --- | --- | --- |

My colleagues encourage me to use the EHR

1----------------- 2 ----------------- 3 -------------------- 4 --------------------- 5 ----------------- 6 ----------------- 7

| Strongly disagree | Disagree | Somewhat disagree | Neither disagree nor agree | Somewhat agree | Agree | Strongly agree |
| --- | --- | --- | --- | --- | --- | --- |

I found the help I needed to use the EHR from my colleagues

1----------------- 2 ----------------- 3 -------------------- 4 --------------------- 5 ----------------- 6 ----------------- 7

| Strongly disagree | Disagree | Somewhat disagree | Neither disagree nor agree | Somewhat agree | Agree | Strongly agree |
| --- | --- | --- | --- | --- | --- | --- |

The EHR quality is better than I expected

1----------------- 2 ----------------- 3 -------------------- 4 --------------------- 5 ----------------- 6 ----------------- 7

| Strongly disagree | Disagree | Somewhat disagree | Neither disagree nor agree | Somewhat agree | Agree | Strongly agree |
| --- | --- | --- | --- | --- | --- | --- |

The EHR usability is better than I expected

1----------------- 2 ----------------- 3 -------------------- 4 --------------------- 5 ----------------- 6 ----------------- 7

| Strongly disagree | Disagree | Somewhat disagree | Neither disagree nor agree | Somewhat agree | Agree | Strongly agree |
| --- | --- | --- | --- | --- | --- | --- |

The EHR usefulness in my practice is better than I expected

1----------------- 2 ----------------- 3 -------------------- 4 --------------------- 5 ----------------- 6 ----------------- 7

| Strongly disagree | Disagree | Somewhat disagree | Neither disagree nor agree | Somewhat agree | Agree | Strongly agree |
| --- | --- | --- | --- | --- | --- | --- |

The quality of the EHR support is better than I expected

1----------------- 2 ----------------- 3 -------------------- 4 --------------------- 5 ----------------- 6 ----------------- 7

| Strongly disagree | Disagree | Somewhat disagree | Neither disagree nor agree | Somewhat agree | Agree | Strongly agree |
| --- | --- | --- | --- | --- | --- | --- |

I am generally satisfied with my experience with the use of EHR

1----------------- 2 ----------------- 3 -------------------- 4 --------------------- 5 ----------------- 6 ----------------- 7

| Strongly disagree | Disagree | Somewhat disagree | Neither disagree nor agree | Somewhat agree | Agree | Strongly agree |
| --- | --- | --- | --- | --- | --- | --- |

I am satisfied with the improvements made to the EHR

1----------------- 2 ----------------- 3 -------------------- 4 --------------------- 5 ----------------- 6 ----------------- 7

| Strongly disagree | Disagree | Somewhat disagree | Neither disagree nor agree | Somewhat agree | Agree | Strongly agree |
| --- | --- | --- | --- | --- | --- | --- |

I am satisfied with my general computing experience at the hospital

1----------------- 2 ----------------- 3 -------------------- 4 --------------------- 5 ----------------- 6 ----------------- 7

| Strongly disagree | Disagree | Somewhat disagree | Neither disagree nor agree | Somewhat agree | Agree | Strongly agree |
| --- | --- | --- | --- | --- | --- | --- |

If given the choice, I will continue to use the EHR (paperless) system

1----------------- 2 ----------------- 3 -------------------- 4 --------------------- 5 ----------------- 6 ----------------- 7

| Strongly disagree | Disagree | Somewhat disagree | Neither disagree nor agree | Somewhat agree | Agree | Strongly agree |
| --- | --- | --- | --- | --- | --- | --- |

I will continue to use the EHR in the future

1----------------- 2 ----------------- 3 -------------------- 4 --------------------- 5 ----------------- 6 ----------------- 7

| Strongly disagree | Disagree | Somewhat disagree | Neither disagree nor agree | Somewhat agree | Agree | Strongly agree |
| --- | --- | --- | --- | --- | --- | --- |

I want to improve my EHR skills

1----------------- 2 ----------------- 3 -------------------- 4 --------------------- 5 ----------------- 6 ----------------- 7

| Strongly disagree | Disagree | Somewhat disagree | Neither disagree nor agree | Somewhat agree | Agree | Strongly agree |
| --- | --- | --- | --- | --- | --- | --- |
